# Editorial: Brain Cancers: New Perspectives and Therapies

**DOI:** 10.3389/fnins.2022.857408

**Published:** 2022-02-14

**Authors:** Elisa Roda, Maria Grazia Bottone

**Affiliations:** ^1^Toxicology Unit, Laboratory of Clinical and Experimental Toxicology, Pavia Poison Centre, National Toxicology Information Centre, Istituti Clinici Scientifici Maugeri IRCCS, Pavia, Italy; ^2^Department of Biology and Biotechnology “L. Spallanzani, ” University of Pavia, Pavia, Italy

**Keywords:** brain tumors, glioblastoma, novel therapies, chemotherapeutic medicines, tumor resistance

Brain diseases come in many different forms. It is estimated that these pathologies affect the lives of 1 in 6 people, and cost over a trillion dollars in annual treatment. The major categories of brain diseases include diverse brain cancers. Brain tumors are the most primitive, invasive and malignant in humans with poor survival after diagnosis (Mckinney, 2004; Laquintana et al., 2009). Although in recent years, numerous studies have been carried out to identify novel therapeutic protocols and tumor molecular markers capable to predict survival and response to treatment, the life expectancy of neuro-oncological patients is still very limited (24–36 months) (Aldape et al., 2019; Liang et al., 2020).

About 33% of all brain tumors are gliomas, accounting for about 80% of the total malignant central nervous system (CNS) tumors in adults (Hanif et al., 2017). Glioma is a broad category of glial brain and spinal cord tumors which originate in the glial cells that surround and support neurons in the brain, including astrocytes, oligodendrocytes, and ependymal cells. Among these, glioblastoma (GBM) is one of the most common and aggressive primary brain tumors (van Tellingen et al., 2015; Davis, 2016; Taylor et al., 2019; Birzu et al., 2021), characterized by diffuse infiltration of the adjacent brain parenchyma and development of drug resistance to standard treatment (Chen et al., 2018; Shergalis et al., 2018). So far, GBM remains associated with an extremely aggressive clinical course, and only 0.05–4.7% of patients survive 5 years from diagnosis (Ostrom et al., 2018). Cellular pleomorphism with nuclear atypia, high mitotic activity, and microvascular proliferation distinguish GBM from other lower-grade gliomas (Hambardzumyan and Bergers, 2015). In addition, the inter- and intra-patient tumor heterogeneity causes several obstacles, limiting the improvement of an early diagnosis and treatment protocols.

The tumor microenvironment (TME) plays a crucial role in mediating tumor progression and invasiveness, contributing to brain tumor aggression and poor prognosis (Di Cintio et al.; Yekula et al., 2020). Recent studies showed that differentiated tumor cells may have the ability to dedifferentiate acquiring a stem-like phenotype in response to microenvironment stresses such as hypoxia. Acidic extracellular pH and nitric oxide were also shown to be involved in stemness preservation (Dahan et al., 2014). Currently, the standard of care consists of surgical resection followed by radiotherapy (RT) and concomitant and adjuvant chemotherapy. Despite this aggressive treatment regimen, the median survival is only around 15 months, and the 2-year survival rate is only 26.5% (von Neubeck et al., 2015; Chen et al., 2018). Indeed, due to the location of gliomas origin and infiltrative growth (Urbańska et al., 2014), complete surgical resection of the tumor is often not possible other than with a high risk of neurological damages for the patient (Goldbrunner et al., 2018). Treating patients with primary brain tumors and brain metastases can be challenging. This is primarily due to the presence of the blood–brain barrier (BBB), posing an obstacle to overcome for most systemic treatments (van Tellingen et al., 2015; Brahm et al., 2020). Despite initial benefits, chemotherapy, using conventional agents, e.g., alkylating agents such as temozolomide, platinum-based drugs, or VEGF inhibitors (Dasari and Tchounwou, 2014; Pérez et al., 2019; Senbabaoglu et al.; Strobel et al., 2019), is often associated with severe systemic toxicity, which occurs especially after long-term treatment (Karasawa and Steyger, 2015; Chovanec et al., 2017). Among these adverse side effects, neurotoxicity assumed increasing clinical importance as it is dose-cumulative and becomes limiting in long-lasting therapies, and also to the severe side effects (Chovanec et al., 2017; Staff et al., 2019). Therefore, high-grade gliomas or GBM are currently considered incurable and all patients inevitably experience and succumb to tumor recurrence, highlighting the urgent need to identify, validate and apply new therapeutic options (Ravanpay et al., 2019; Taylor et al., 2019; Maggs et al.; Ghouzlani et al., 2021).

This Frontiers Research Topic Proposal on “*Brain Cancers: New Perspectives and Therapies*” joined contributions from scientists and physicians who investigate on etiopathogenesis and treatment of brain cancers. In fact, studies exploiting the existing link between enhancing the knowledge of cellular and molecular pathways involved in the onset/progression of these pathologies and the development of innovative therapies, improving patient prognosis and quality of life, need further in-depth investigations.

The published articles are based on neuro-oncological research and deal with proposing novel effective therapeutic strategies, focusing on different targets and aspects typical of brain tumors: tumor heterogeneity and microenvironment, cancer cell response to new chemotherapeutics and innovative radiotherapy treatments settings (often tested in combined protocols), immune-mediated gene therapies, which may involve blockade of immune checkpoint inhibitors, and other targeted therapies such virotherapy, CAR-T cells, dendritic cells' vaccines, or nanoparticle-mediated vaccination technologies (Alghamri et al.; Brandalise et al.; Chen et al.; Di Cintio et al.; Ferrari et al.; Lange et al.; Maggs et al.; Pasi et al.; Senbabaoglu et al.).

The joint mechanisms of neuro-inflammation, tumor microenvironment and BBB leakage status, which have been shown to trigger the tumor onset, invasion and progression, often mediated by the deregulation of a number of channel proteins and ion pumps (Brandalise et al.), have been also explored as promising targets for personalized pharmacological interventions (Alghamri et al.; Di Cintio et al.; Lee et al., 2020). Another exploited key mechanism is cell death, a crucial multifaceted process dependent on signal transduction pathways, in which several Hsp90 client proteins, frequently abnormally expressed, may be involved (Cao et al.; Chen et al.). It widely accepted that in cancer cells, particularly in gliomas cells, cell death pathways can be deactivated or defective for various causes, thus promoting cancer formation, proliferation, invasiveness, and even the induction of resistance to the drugs treatment. Particular effort has been devoted to the repositioning of old drugs as potent therapeutics for GMB and/or to exploit the combined effects of novel drugs in synergism with different irradiation protocols (Chen et al.; Lange et al.; Ferrari et al.; Pasi et al.).

In summary, clinical evidences highlights the urgent medical need to further comprehend and delineate the complex mechanisms/interactions between cancer cells, immune cells, tumor stroma, resident healthy brain cells, and tumor vasculature, to develop innovative effective treatment strategies through the identification of novel targets. A multidisciplinary approach, taking into consideration all brain tumors aspects, including the modulation of the communication processes between cancer niche and tumor microenvironment and also the potential reactivation of defective cell death mechanisms, can currently be considered as a promising strategy.

This Frontiers Research Topic had the ultimate goal to apply new knowledges coming from multitiered approaches, to identify novel effective therapeutic strategies to be used in the field of clinical neuro-oncology, to improve the patient prognosis and quality of life, also reducing adverse side effects due to conventional treatments, in view of a focused, personalized medicine. The published contributions may play a crucial role, laying the groundwork to translate the experimental findings to clinical setting, turning them into new clinical therapeutic protocols, facing the challenges in this field and developing new healing perspectives.

## Author Contributions

Both authors equally contributed to the work, giving a substantial, direct, and intellectual contribution, and they both approved the work for publication.

## Conflict of Interest

The authors declare that the research was conducted in the absence of any commercial or financial relationships that could be construed as a potential conflict of interest.

## Publisher's Note

All claims expressed in this article are solely those of the authors and do not necessarily represent those of their affiliated organizations, or those of the publisher, the editors and the reviewers. Any product that may be evaluated in this article, or claim that may be made by its manufacturer, is not guaranteed or endorsed by the publisher.
